# The transboundary nature of the world’s exploited marine species

**DOI:** 10.1038/s41598-020-74644-2

**Published:** 2020-10-21

**Authors:** Juliano Palacios-Abrantes, Gabriel Reygondeau, Colette C. C. Wabnitz, William W. L. Cheung

**Affiliations:** 1 Institute for the Oceans and Fisheries, University of British Columbia, Vancouver, BC Canada; 2 Department of Ecology and Evolutionary, Yale University, New Haven, CT USA; 3 Stockholm Resilience Centre, Stockholm University, Stockholm, Sweden; 4 Center for Ocean Solutions, Stanford University, Stanford, CA USA

**Keywords:** Ecosystem services, Sustainability, Biogeography

## Abstract

Regulatory boundaries and species distributions often do not align. This is especially the case for marine species crossing multiple Exclusive Economic Zones (EEZs). Such movements represent a challenge for fisheries management, as policies tend to focus at the national level, yet international collaborations are needed to maximize long-term ecological, social and economic benefits of shared marine species. Here, we combined species distributions and the spatial delineation of EEZs at the global level to identify the number of commercially exploited marine species that are shared between neighboring nations. We found that 67% of the species analyzed are transboundary (n = 633). Between 2005 and 2014, fisheries targeting these species within global-EEZs caught on average 48 million tonnes per year, equivalent to an average of USD 77 billion in annual fishing revenue. For select countries, over 90% of their catch and economic benefits were attributable to a few shared resources. Our analysis suggests that catches from transboundary species are declining more than those from non-transboundary species. Our study has direct implications for managing fisheries targeting transboundary species, highlighting the need for strengthened effective and equitable international cooperation.

## Introduction

Distributions of marine species around the world are not constrained by human-made boundaries; rather they are shaped by biotic and abiotic factors as well as species’ evolutionary history^1,2^. A species can be widely distributed (cosmopolitan) or endemic^2^. Fisheries management is predicated on the definition of “stocks”, delineated, in most cases by human-made spatial boundaries that often do not correspond to biologically-meaningful population units^3,4^. The development of Exclusive Economic Zones (EEZs) under Part V of the United Nations Convention on the Law of the Seas (UNCLOS) in the early 80s^6^, for instance, extended political boundaries from 12 to 200 nautical miles to give coastal nations property rights over marine resources^5^. However, EEZ boundaries cut across the distribution of many species, creating shared stocks between nations^6^. Shared stocks can be classified into three non-exclusive categories; “transboundary”—stocks that cross the EEZs of two or more bordering coastal states; “straddling”—stocks that cross neighboring EEZs and the adjacent high seas; and “highly migratory”—stocks that cross non-neighboring EEZs and the high seas (mainly tunas)^7^. Our study focuses on the “transboundary” nature of shared species exploited by fisheries operating within EEZs.

Theory and empirical evidence have shown that fisheries targeting resources that straddle political boundaries complicate fisheries management and potentially reduce the effectiveness of policies to achieve their stated objectives^8,9^. For instance, climate-driven changes in species distributions have led to conflicts between nations, driven, at least partly, by changes in the proportion of captures^8^, quota allocation, and fishery newcomers^10^. Moreover, climate change is likely to exacerbate such conflicts and presents new challenges for political relations between neighboring countries^11^ and fisheries management^12^. Therefore, having an accurate understanding of the distribution and scale of transboundary and straddling fish stocks as well as associated fisheries is important to inform their sustainable management, particularly under climate change.

Forty years after the formal adoption of UNCLOS^6^ and the subsequent 1995 United Nations Fish Stock Agreement for the cooperation on the management and conservation of straddling and highly migratory resources^13^, accurate estimates of the number of exploited marine species shared by neighboring nations are still unavailable. An informed guess, based on limited biogeographical data, suggested that there are approximately 500–1500 exploited transboundary stocks^14^. A recent literature review included 344 shared taxa and their historical contributions to fisheries^15^. However, these studies did not consider species’ biogeography in the quantification of transboundary stocks. Here, we aim to estimate the number of exploited marine species shared by neighboring countries and determine their contribution to global and regional catches as well as fisheries revenue. Moreover, we categorize species according to their catch trends while identifying differences among species based on habitat preference. We hypothesize that the methodological constraints of previous studies resulted in an underestimation of the number of transboundary species and their contribution to global catch and revenue.

We overlaid the known distribution of 938 commercially valuable marine species responsible for an average of 96.5% of global EEZ catches between 2005 and 2014, and 280 EEZs of 198 coastal countries (see Methods). We define a ‘stock’ unit as a species in an EEZ, instead of a genetically or morphological distinct unit^2^, due to the lack of such biological information being available for almost all the species included in this study^15^. While we acknowledge that species could have multiple stocks within an EEZ, many fisheries within a country or EEZ are managed at the species instead of stock level (or even as groups containing multiple species). For example, shrimp (e.g., *Litopenaeus stylirostris* or *Farfantepenaeus californiensis*) along the Pacific coast of Mexico^16^ and hammerhead shark (*Sphyrna zygaena*) in Peru^17^ are managed at the species level, yet include multiple populations exploited by each country’s fisheries. Moreover, recent research shows connectivity across fish stocks through larval dispersal^18^ and adult migration^19–21^, although considerable level of uncertainty exists at different life stages^22,23^. For this analysis we only considered shared species between neighboring EEZs, rather than the species’ extended distribution (e.g., we did not include the high seas). We rely on multiple data sources including occurrence, distribution models and catch data, and only consider a species to be present in a grid cell if all data sources showed positive occurrence (see Methods).

## Results and discussion

**Figure 1:**
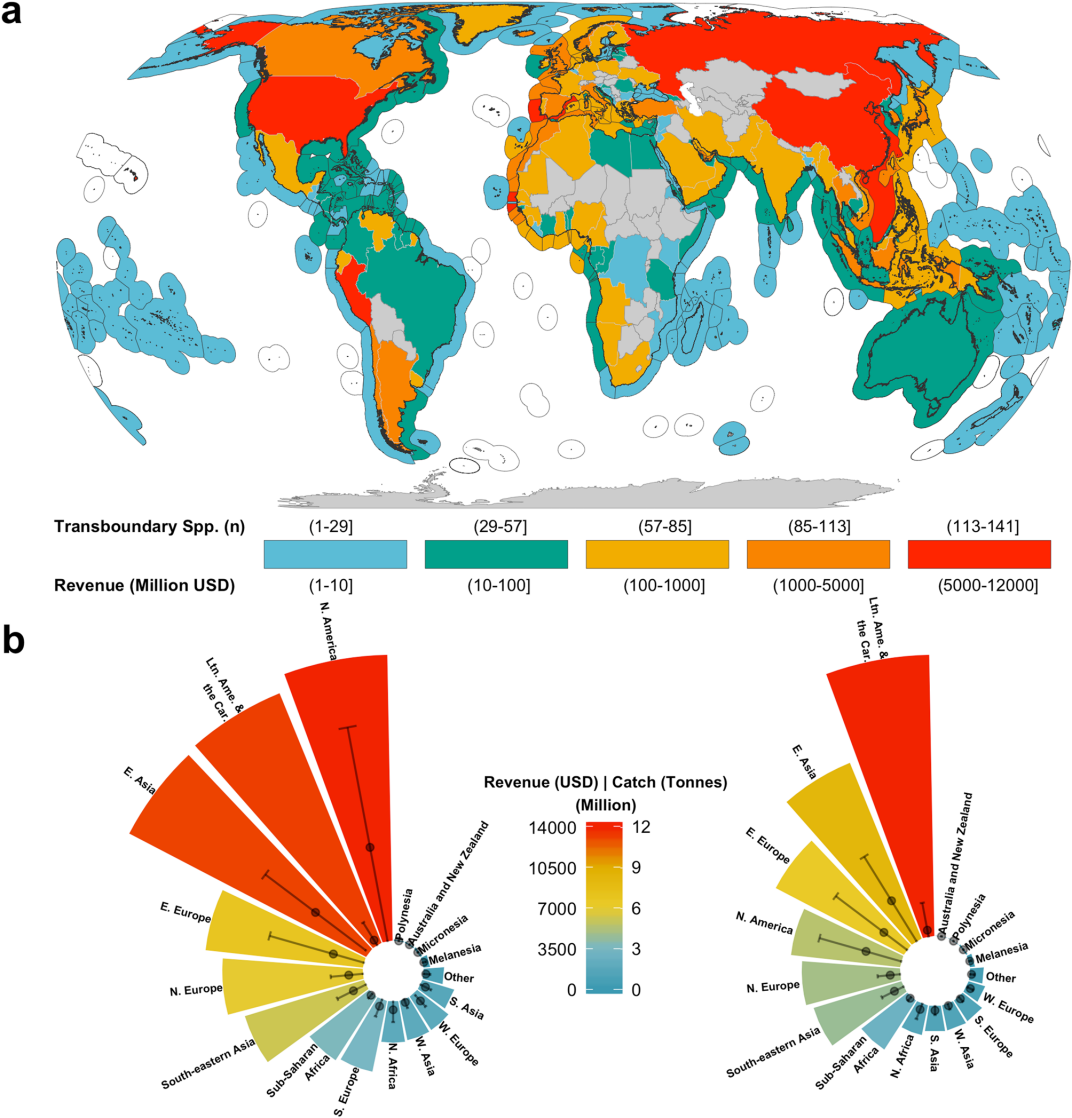
Number of transboundary species and their contribution to global fisheries catch and revenue. (**a**) The number of species and amount of revenue are represented by color coding of EEZs and land polygons, respectively. (**b**) Contribution of transboundary species to regional revenue (left) and catch (right). Regions classified according to the United Nations sub-regions: *E.* Eastern, *N.* Northern, *S.* Southern, *W.* Western, *Ltn. Ame. & the Car.* Latin America and the Caribbean. Points = regional mean ± sd. Revenue in 2019 real USD. Figure created using R version 3.5.2. Land and EEZ shapefiles from Natural Earth (www.naturalearthdata.com) and *Sea Around Us* (www.seaaroundus.org), respectively.

We identified 633 exploited transboundary species worldwide (67.5% of the 938 species analyzed), almost double previous estimates^15^. Between 2005 and 2014, national fleets targeting these transboundary species within EEZs caught an annual average of 48.5 million tonnes, representing 82.3% of EEZ-derived catches reconstructed by the *Sea Around Us* at the species level (Fig. 1a). These catches generated a yearly average of USD 77,591 million in fishing revenue (78.5% of global fishing revenue) over the same time period. Our findings are considerably higher than the previous estimates of 34.2 million tonnes in catches and USD 40,187 million (in 2019 value) in fishing revenue from shared stocks^15^.When we re-estimated transboundary species’ catches using data consistent with those used in previous studies (FAO global reported data in year 2006), the 633 transboundary species identified here accounted for 40.4 million tonnes of annual catches (i.e., 18% higher than previously estimated). Our results suggests that the contribution of transboundary species to global catch and fishing revenue might previously have been underestimated due to an incomplete understanding of the transboundary nature of marine fished species. The 305 non-transboundary species (termed as ‘discrete’ species here, see Methods—Determining transboundary species trait) accounted for a much smaller proportion of total catch and revenue; 2.8 million tonnes and USD 4,282 million annually respectively, on average, between 2005 and 2014. These results underscore the importance of transboundary species at the global level.

In many cases, according to our categorization criteria, a transboundary species can be distributed in multiple EEZs but only counted as transboundary in a subset of EEZs (see Methods). For example, the distribution of Peruvian anchoveta (*Engraulis ringens*) spans the EEZs of Peru, Chile, and Ecuador. However, our study only considered the stocks in Peru and Chile as transboundary^24^. Anchoveta in Ecuadorian waters only include a small proportion of the shared distribution range (spatial threshold between Ecuador and Peru < 10%; see Methods—Criteria 3) and thus do not meet our criteria for consideration as a transboundary stock. A situation similar to the example of Peruvian anchoveta in Ecuadorian waters is common amongst the transboundary species identified in this study. Overall, 590 of the 633 transboundary species have stocks in EEZs that do not meet our criteria for consideration as transboundary stock. The annual average contribution from all excluded stocks of transboundary species to fisheries (e.g., Ecuador’s anchoveta catch) between 2005 and 2014 was 10.8 million tonnes, representing USD 19,243 million in fishing revenue over the same time period.

At a regional level, we found that transboundary species are particularly economically important for Northern America (average country revenue = USD 4,680 $\pm$ 6,000 million) and Eastern Asia (USD 3,779 $\pm$ 3,093 million) (Fig. 1b). The estimated per country revenues from transboundary species in these two regions is significantly different from other regions (one-way ANOVA; *DF*(16,165) = 5.081, *p* < 0.001, $\alpha$ = 0.05). China (USD 7,284 million) and the USA (USD 11,604 million) contribute 55% and 82% to the annual average revenue from 2005 to 2014 in Eastern Asia and Northern America, respectively. In addition to China and the USA, Russia (USD 7,379 million), Peru (USD 6,044 million) and Japan (USD 3,907 million) were among the top five countries with the most fishing revenue generated from transboundary species between 2005 and 2014 (Fig. 1a). These five nations were responsible for 41% of the yearly global fisheries revenue from transboundary species.

**Figure 2:**
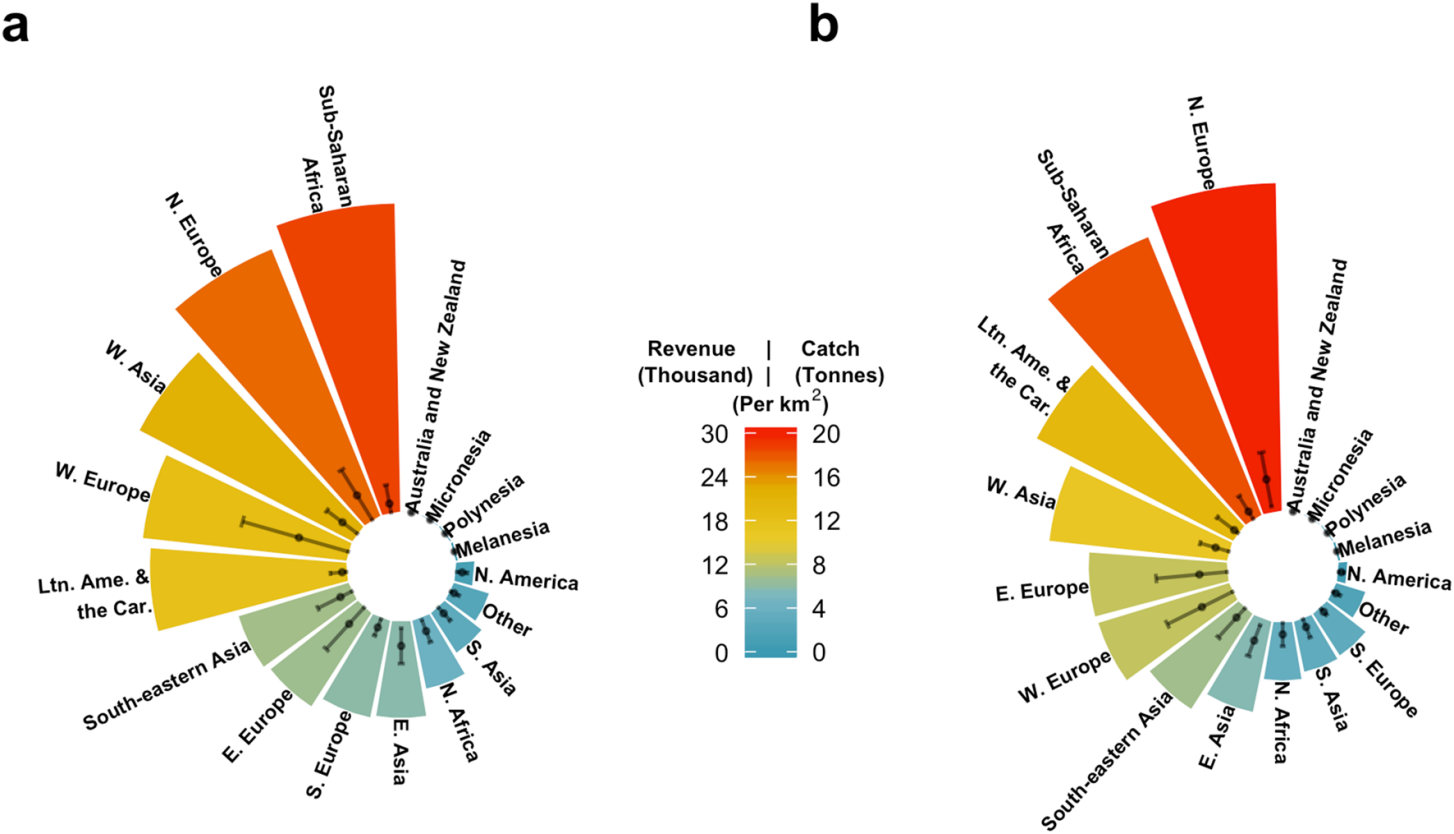
Weighted benefits of transboundary species by ${\mathrm{km}}^{2}$ and UN sub-region. (**a**) Revenue in thousand 2019 USD. (**b**) Catch in tonnes. Points = sub-region mean  $\pm$ s.d. by country. Figure created using R version 3.5.2. Abbreviations as in Fig. 1.

Peru and Russia, having the two largest fisheries by total catch in the world^25^, were each responsible for over 5.8 million tonnes of transboundary species catch annually, on average, between 2005 and 2014 (Supplementary Fig. S1). Peru’s catches consisted mainly of Peruvian anchoveta (*Engraulis ringens*) and accounted for 79% of the national transboundary species production. Peru and Chile recently signed an agreement to work towards standardized stock assessments through coordinated management of the southern anchoveta stock^26^. A similar management agreement was signed by Russia, Japan and the USA over shared Alaskan pollock (*Theragra chalcogramma*) in the Bering Sea in 1988^27^. Transboundary species also make large contributions to fisheries in Eastern Asia (one-way ANOVA; *Df*(16,158) = 2.265, *p* = 0.005, $\alpha$ = 0.05). China, the world’s top fish producer^25^, obtains one third (5.1 million tonnes) of its total catches from transboundary species, followed by Japan (1.8 million tonnes) and South Korea (1.06 million tonnes). Differences in the regional importance of transboundary fisheries can also be illustrated in terms of catch-revenue over the area ($k{m}^{2}$) of the EEZ (Fig. 2). As an example, the aggregated EEZ area for all Northern European countries where transboundary species are present is 3.3 million $k{m}^{2}$, the ${6}^{th}$ smallest of the 17 groups analyzed (Supplementary Table S1). However, it had the second highest average revenue (USD 26.1 thousand per $k{m}^{2}$) and the highest average catch (19.9 tonnes per $k{m}^{2}$) of transboundary species per EEZ area between 2005 and 2014. At the country level, countries from Western Europe accrued significantly more revenue from transboundary fisheries per $k{m}^{2}$ than any other country (one-way ANOVA, *Df*(16,165) = 3.267, *p* < 0.001, $\alpha$ = 0.05; Tukey’s post hoc test *p* $\le$ 0.05; Fig. 2).

**Figure 3:**
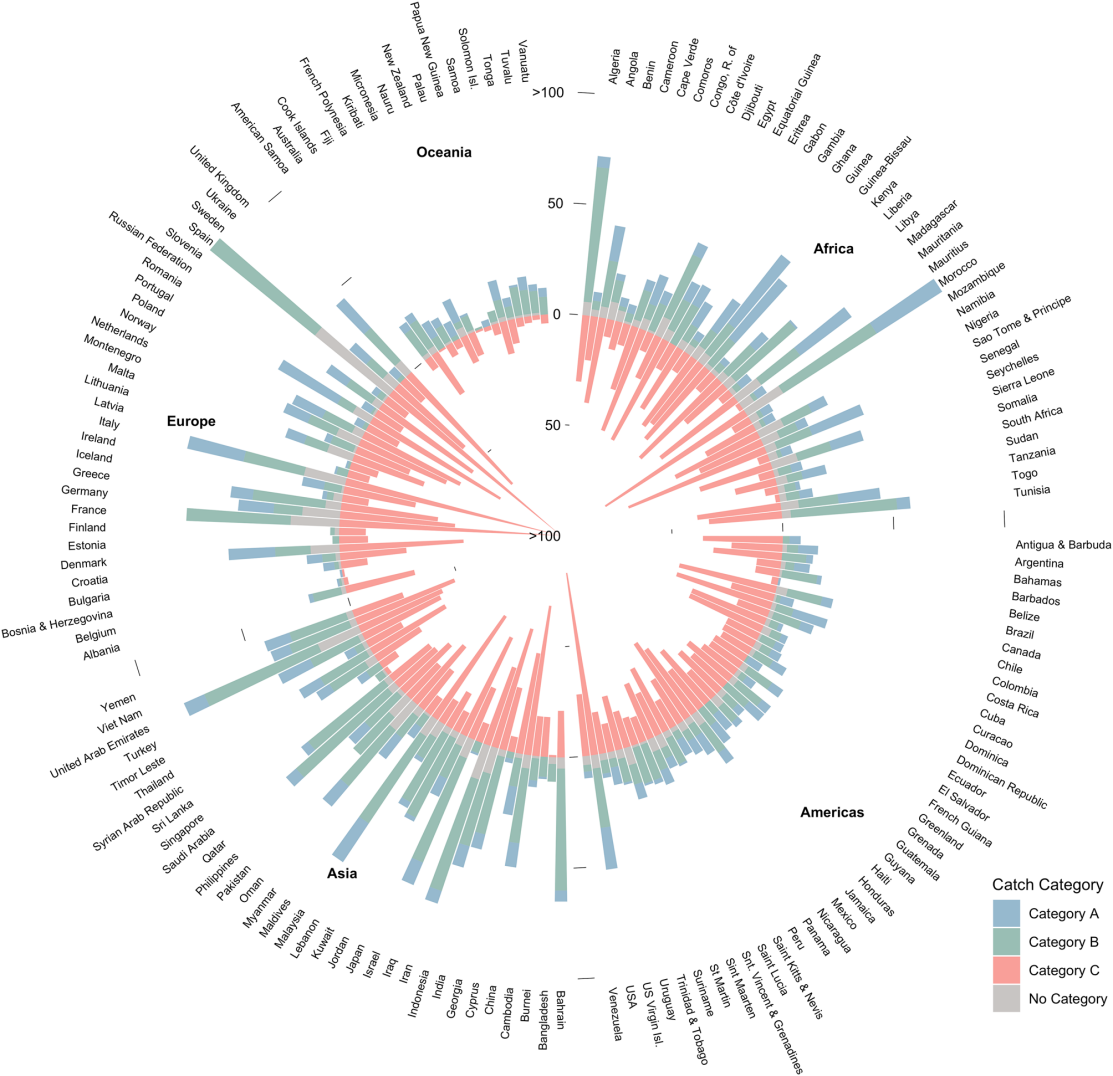
Number of transboundary species by catch trend and EEZ. Category A, Increasing; Category B, Constant; Category C, Decreasing. “No Category” reflects species with less than 10 years of catch data and/ or less than 5 consecutive years of catch data. Only showing first 100 species. Figure created using R version 3.5.2.

We determined the catch trend of each species within each EEZ, classifying them as increasing (Category A), constant (Category B) or decreasing (Category C) (Fig. 3, Supplementary Fig. S2). While previous studies have demonstrated that catch trends can be used to infer whether a stock is healthy, re-building, over-exploited or collapsed^28^, several factors can influence stock status. Our intention here is to examine where the catch trends of transboundary species differ from non-transboundary species between 2005 and 2014 relative to historic catch since 1951 (see Mhods). We found significant differences for all catch trend categories for transboundary species (one-way ANOVA, *Df*(2,459) = 47.94, *p* < 0.001, $\alpha$ = 0.05; Tukey’s post hoc test *p* $\le$ 0.001), and no significant differences in catch trend categories for discrete species (one-way ANOVA, *Df*(2,106) = 1.885, *p* = 0.157; $\alpha$ = 0.05). We also found significant differences in catch trends when directly comparing transboundary to discrete species categories (MANOVA, *Df*(2,459) = 19.001, *p* < 0.001). Overall, transboundary species only targeted by one country are generally less likely to have a decreasing catch trend compared to instances where the shared species is fished by neighboring countries (Supplementary Table S2).

Empirical analysis suggests that in most cases, management of transboundary species will yield better outcomes in terms of fish catches when nations cooperate^8^. Yet, cooperation can be a complex process^29,^ and in specific cases joint management might not be the best strategy^30^. Examples of successful joint management include agreements between Norway and Russia over Atlantic cod (*Gadus morhua*)^31^ and Namibia and South Africa over hake (*Merluccius spp*)^32^. Lack of collaboration over shared stocks may threaten stock sustainability, reduce the future profitability potential of the fishery, and result in conflict between neighboring nations^10,33^.

Transboundary fisheries are important to a number of countries with notorious fisheries-related conflicts, including Canada, the USA, the European Union (EU) and Russia^34^. For example, since 2007, the EU, Norway, Iceland, and the Faroe Islands (Denmark) have been at odds over the size and relative allocation of the total allowable catch for Atlantic mackerel (*Scomber scombrus*) due to the species' climate-driven shift in distribution^10^. Atlantic Mackerel is a transboundary species that straddles into the high seas. Among the countries involved in the 2007 fisheries dispute, Atlantic mackerel contributed an annual average (between 2005 and 2014) of 598.4 thousand tonnes (8.19%) in total catch and USD 850.2 million (7.17%) in total fishing revenue. Climate change is expected to continue changing the distribution and shared proportion of fish stocks among countries, resulting in the emergence of new transboundary species^11^, and disappearance of some species from EEZs^35^. Exploring the detailed effects of climate change on the distribution of shared species is key to the development of local adaptation methods that can anticipate negative impacts to sustainability. For example, understanding how climate change will modify the proportion of transboundary species shared by neighboring EEZs, the time-frame over which such changes will happen^36^, and the economic consequence of such effects can inform the development of more anticipatory and climate-resilient international treaties, improving fisheries management^37^.

**Figure 4:**
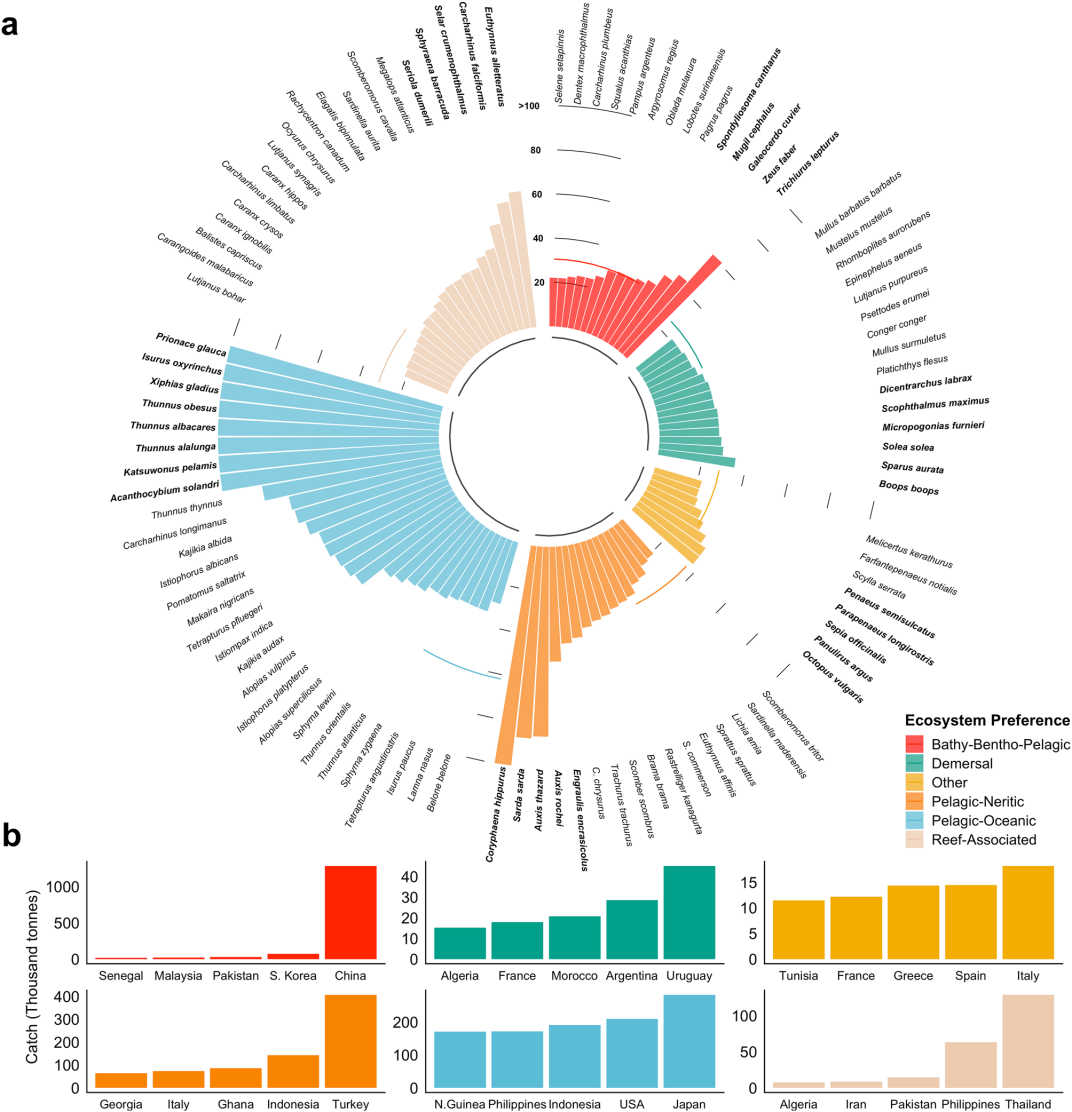
Number of EEZs shared by transboundary species. (**a**) Number of EEZs by species organized according to ecosystem preference as defined by FishBase. Showing only species that share > 20 EEZs. (**b**) Average catch between 2005 and 2014 for top five countries capturing the top five shared species for each ecosystem preference category (color coding is as shown in legend in a). Note that there could be > 5 species due to similar sharing values. The category “Other” consists of species that have no ’ecosystem preference’ classification in FishBase. Figure created using R version 3.5.2.

Most marine fish species occur in tropical and subtropical waters around the world^2,38^, from highly migratory species associated with pelagic-oceanic ecosystems like tunas (*Thunnus sp.*), to less mobile reef-associated species like greater amberjack (*Seriola dumerili*), and species found in demersal ecosystems like gilthead seabream (*Sparus aurata*). Species associated with  pelagic-oceanic ecosystems are the only group whose EEZ range (i.e., the number of EEZs where the species occur as transboundary) is significantly different from other groups (one-way ANOVA; *DF*(5,597) = 53.82, *p* < 0.001, $\alpha$ = 0.05; Tukey’s post hoc test *p* < 0.05), with a median of 40 EEZs per species. The median for species of all other ecosystem preferences is close to, or less than, 20, as many of these species have a narrower distribution or are less mobile (Fig. 4a). This result is likely an effect of the broad distribution that many of the species with preference for pelagic-oceanic ecosystems have. Many pelagic-oceanic species are highly migratory and straddle across EEZs while crossing the high seas. Thus, the number of non-neighboring EEZs sharing a highly migratory species can be over 100, as in the case of bigeye tuna (*Thunnus obesus*) (Fig. 4a). Due to their vast migration patterns and presence in areas beyond national jurisdiction, many highly migratory species are managed by Regional Fisheries Management Organizations (RFMOs).

Many transboundary species are not considered highly migratory, but are still shared by numerous neighboring EEZs (e.g., garfish *Belone belone*) (Fig. 4a). In addition, many fish stocks have meta-populations that are connected through larval dispersal with ‘source’ populations potentially supporting ‘sink’ populations thousands of kilometers away^18^. For example, while coral reef-associated species were found to share fewer neighboring EEZs than other species (Fig. 4a), coral regions often share multiple species through larval connectivity^39^ and adult movement^19^. However, it is important to acknowledge the uncertainty in the connectivity of marine populations at different life stages from larvae^22^ to adults^23^. In some areas like the Caribbean and the Western Indian Ocean, among other regions, transboundary marine protected areas have been identified as potential tools to support fisheries and conservation goals^19,40^. The effective management of coral reef species is critical to many coastal communities, as they tend to be highly dependent on subsistence fishing for food and nutrition security, as well as livelihoods^41,42^. For instance, a number of countries with the largest catches of transboundary reef and pelagic-oceanic associated species (Fig. 4b) are also associated with some of the highest rates of fish consumption^25^. In the Philippines, both pelagic and reef fishes contribute substantially to both local food and nutrition security, as well as livelihoods^43^.

## Conclusions

Our study identified species currently shared by neighboring coastal nations and highlights the importance of these species’ contribution to global catch and revenue derived from wild fisheries. Our results show that captures and revenues from shared species are substantially higher than previously estimated^15^ and also much greater than catches and revenues obtained from discrete species. This result highlights the importance of transboundary fisheries and their potential contribution to food and nutrition security, as well as livelihoods. Moreover, we show significant differences in the catch trends of transboundary and discrete species, suggesting a need to improve the management of transboundary fisheries. Previous work has highlighted that collaboration is key to better outcomes for shared marine resources^8^. Identifying existing transboundary species is the first step towards cooperative joint management frameworks that are precautionary, strive for sustainability, and can be flexible to accommodate the uncertain future driven by climate change.

## Materials and methods

### Databases of species’ geographic distribution

The *Sea Around Us* has reconstructed global fisheries catches from 1950 to 2014, identifying commercial marine fish and invertebrates as well as fishing regions^44^. We used the *Sea Around Us* reconstructed species list to determine the number of transboundary marine species exploited by fisheries within each of the world’s EEZs. The total number of species analyzed was 938, representing 67% of identified species by the *Sea Around Us* and accounting for 96.52% of the catch identified at the species’ level. To determine the current distribution of exploitable marine species, we used four data sources of species-distributions: (*i*) occurrence data, (*ii*) an Ensemble Environmental Niche Model (ENMs), (*iii*) a life-history-based distribution model, and (*iv*) fisheries catch data (Table 1). Each source represents a different method of estimating the distribution of a given species, thus providing a more robust result than an analysis focused on a single data source. Only commercially fished species with data from all four sources were included in our analysis.

**Table 1:** Summary of data sources to estimate species’ distributions. All data has a resolution 0.5°latitude × 0.5°longitude. All data are publicly available (see "Acknowledgments").

Source	Abbreviation	Main method	Sources
Occurrence data	Occurrence	Occurrence data from multiple sources	Reygondeau^38^
Ecological niche model	ENM-Nereus	Environmental niche model based on environmental variables and different model algorithms	Asch et al^.^^48^, Reygondeau^38^
Species distribution model	SDM-SAU	Species distribution model based on species traits	Close et al*.*^49^, Pauly^50^
Catch data	Catch-SAU	Spatial catch allocation based on country-by-country reconstructions	Zeller et al.^44^

#### Occurrence data

Occurrence data was collected by Reygondeau^38^ from five publicly available repositories: FishBase (https://fishbase.org), the Global Biodiversity Information Facility (GBIF; https://www.gbif.org/), the Ocean Biogeographic Information System (OBIS; https://obis.org/), the Intergovernmental Oceanographic Commission (IOC; https://ioc-unesco.org), and the International Union for Conservation of Nature (IUCN; https://www.iucn.org/technical-documents/spatial-data)38.

#### Distribution models

In addition to occurrence data, we used two different methods to estimate species distributions, hereafter referred to as Ecological Niche Model-Nereus (ENM-Nereus) and Species Distribution Model-SAU (SDM-SAU). Although they use the same occurrence and environmental data, the models are structurally different, complementing each other and providing robustness to the results.

The ENM-Nereus consists of a multimodel approach based on a Bioclim and a Boosted Regression Tree model^45^, a Maxent model^46^, and a Non-Parametric Probabilistic Ecological Niche Model^47^. Environmental variables utilized in the models include sea surface temperature, surface pH, surface oxygen concentration, and vertically integrated (0–100 m) net primary production (NPP)^48^. Global environmental conditions were averaged for the 30-year climate normal period of 1970–2000 and averaged for three Earth System Models developed by the Geophysical Fluid Dynamics Laboratory (GFDL—https://www.gfdl.noaa.gov/earth-system-model/), the Institute Pierre Simon Laplace (IPSL—www.icmc.ipsl.fr/), and the Max Planck Institute for Meteorology (MPI—www.mpimet.mpg.de/en/science/models/). See^38,48^ for model details.

The SDM-SAU model follows a five-step process based on species-specific life history data, rather than environmental variables^49,50^. For each commercial marine species, the model first uses the FAO major fishing areas and countries’ EEZs to determine a broad distribution. It then uses life history information to delimit its range within the FAO fishing area (e.g., thermal preference, depth limit). The range is delimited even further by expert-review polygons and compared with istributions from AquaMaps^51^,  as well as OBIS and GBIF occurrence data. The model then determines a species’ habitat-preference based on the assumption that the relative abundance of a species is determined by the number of habitats in a grid cell and the species’ distance to each habitat, as well as the importance of the habitat to the total size of the species distribution. Finally, the species equatorial submergence (e.g., the latitudinal region where a species is not seen in between poles) is estimated for each species. See^49,50^ for model details.

#### Catch data

The previous models combine observational data with a series of biotic and abiotic data to determine the probability that a species will be found in a given space at a given time. However, this does not mean that the species in question will actually be there. While the models do use approaches to double-check species occurrences (e.g., ENM-Nereus uses four different species distribution algorithms and SDM-SAU undertakes validation by means of other models), we used a fourth data set to corroborate the models’ outputs. The *Sea Around Us* estimates total reconstructed catches–catches based on all publicly available information sources and including discards, as well as unreported and illegal catches that are not included in available FAO data—for each country. Catches are also spatially allocated on a ${0.5}^{\circ }$ x ${0.5}^{\circ }$ latitude longitude grid^44^. Roughly, the *Sea Around Us* method consist of the following steps. First, it takes each country’s officially reported catch data (e.g., National, FAO or RFMO). Secondly, it uses literature (e.g., peer review, grey literature) to identify missing components (e.g., species, gears) and sources of alternative information for missing components. It then derives country estimates for missing data and creates time series interpolation. Finally, the estimated and official data are aggregated, making up the total reconstructed catch data (see^52^ and^44^ for catch reconstruction and spatial allocation details). We used the *Sea Around Us* catch reconstruction database from 2005 to 2014 as the fourth dataset to estimate transboundary species and to estimate their catch contribution within EEZs. We selected this time frame to investigate the recent (our time frame includes the last decade of available data) contribution of transboundary fisheries to catches and revenue from fisheries and to reduce the uncertainty embedded in the reconstruction process (see key uncertainties, below). Note that in call cases we report the average catch from 2005 to 2014.

### Determining transboundary species trait

We developed a three-criteria methodology to determine whether or not a species can be considered transboundary. Only species that met all criteria at least once were considered as “transboundary”, while species that did not meet the criteria for any EEZ analyzed were considered as “discrete”. Note that in cases where a species met all criteria for some EEZs, but not for other EEZs, these species were still considered as “transboundary”.

### Criteria 1; neighboring EEZs

As mentioned above, we define transboundary species as those marine species that occur within the EEZs of two or more neighboring countries. Hence, according to this criteria a species was only considered as transboundary if it was shared between two neighboring countries, regardless of the species extended distribution. The analysis was undertaken only within the boundaries of the EEZs of coastal states using the *Sea Around Us* shapefile (updated 1 July 2015, available from https://www.seaaroundus.org)—noting that it subdivides the EEZs of 198 coastal states into 280 regions (e.g., Mexico’s EEZ is divided in Mexico Pacific and Mexico Atlantic), including islands territories—and determined the intersections between polygons using the R package *sf*^53^. When estimating transboundary species, we filtered out those shared by EEZs sub-regions (e.g., USA Gulf of Mexico and USA Atlantic), and when aggregating results by country, species that occurred in more than one sub-region were only accounted for once. Species that were present in EEZs that were non-continental territories neighboring other countries were kept as separate (e.g., Argentina and Falkland Islands), but removed in cases where the non-continental territory belonged to the same nation (e.g., Brazil and Fernando de Noronha). Associated states like Puerto Rico and New Caledonia were not considered separately (e.g., Puerto Rico was grouped with the United States and New Caledonia with France).

### Criteria 2; data agreement

We used the occurrence database, the ENM-Nereus model, and SDM-SAU model to determine the presence of each species within each of the world’s ${0.5}^{\circ }$ × ${0.5}^{\circ }$ marine grid cells. All analyses only considered cases with agreement across all databases to obtain a more conservative estimate of transboundary species. Moreover, we assumed that a species was only present in a given grid-cell if it was reported in the Sea *Around Us * catch database. Therefore, all species that were not reported as caught in any single year between the reference years (2005–2014) in a given grid-cell were dropped. This rule assumes that if a commercial species is projected within the EEZ of any fishing country, such a species would have been fished, and thus likely reported at some point over the last decade of data (2005–2014), thereby validating the models and selecting “currently” shared species. We acknowledge that this criteria might limit the global distribution of species therefore resulting in a conservative estimate of transboundary species.

### Criteria 3; spatial distribution

Finally, to have a more robust result and not categorize a species as transboundary based on its presence in a single ${0.5}^{\circ }$ × ${0.5}^{\circ }$ grid cell within an EEZ, we computed an *Area Index*. The *Area Index* represents the proportion of a given species’ overarching shared distribution between neighboring EEZs accounted for by each individual EEZ. We classified a species as transboundary if both neighboring EEZs enclosed over 25% of the species joint shared distribution. While a species that has less than the selected threshold is not considered transboundary in this paper, this threshold can be lowered for a more relaxed result or increased for a more conservative estimate (Supplementary Fig. S2).

### Fisheries trends

We estimated the economic contribution in 2019 real USD of transboundary species for each country using global ex-vessel price data^54^. The database we draw from includes ex-vessel price derived from multiple sources and a structured interpolation method (e.g., similar countries, species) to fill in data gaps^55^. The dataset is harmonized with the *Sea Around Us* catch data to estimate yearly fishing revenue (as ex-vessel price) for all species and EEZs considered in this study (https://www.seaaroundus.org/data/#/feru). We report average fishing revenue derived from fishing activity within global EEZs between 2005 and 2014. We did not include revenue from areas beyond national jurisdiction. We used the monthly average US Consumer Price Index (CPI) according to the U.S. Bureau of Labor Statistic (https://www.bls.gov/cpi/) to standardize the original 2010 real USD value to 2019 real USD.

We used catch data as described above to determine the catch trend of each species within an EEZ. Although this method has previously been used to estimate stock status^56^, the categories presented here are intended to represent catch trends, and not fishing status for each species as many environmental and social-economic factors (e.g., temperature, markets, fishing policies, and fishing effort) affect catches^57,58^. We only assessed species within each EEZ for which at least ten years of data were available between 1951 and 2014 and with at least five consecutive years of data. Three final categories were drawn up for each species depending on catch volume within each EEZ (e.g., present, maximum, and minimum EEZ’s historical catch) and the year (e.g., year of maximum historical catch of the species within that EEZ) (Table 2)^28^. Accordingly, Category A represents fisheries that are registering increases in catch (“increasing”); Category B, species that have a constant catch rate (“constant”); and Category C, species that have registered declines in catch over the last 10 years (“decreasing”). Finally, we report the predominant category over the time period considered.

**Table 2:** Rules to determine the category of each analyzed species.

Categories	Rules
A—Increasing trend	(Year of Catch > Year Post Max. Min. & Post Max Min Catch < (Max Catch*0.10)) & (Catch > (Max Catch*0.10) & Catch < (Max Catch*0.50)) or Year of Catch < Year of Max. Catch & Catch < = (Max Catch*0.50) or Year of Max Catch = Last Year of data)
B—Constant trend	Catch > (Max Catch*0.50)
C—Decreasing trend	Year of Catch > Year of Max. Catch & (Catch > (Max. Catch*0.10) & Catch < (Max. Catch*0.50) or Catch < Max. Catch*0.10
No status	None of the above rules applied

### Statistical analysis

All analyses were run using the statistical software *R version 3.5.2 (2018–12-20)* with the packages *data.table*^59^, *janitor*^60^, *wesanderson*^61^, *rfishbase*^62^, *R.matlab*^63^, *sf*^53^, *sp*^64^, *tidiverse*^65^, *tidytext*^66^, and *zoo*^67^. All code is available at https://github.com/jepa/FishForVisa. All maps were made with Natural Earth data available at https://www.naturalearthdata.com/. We performed a series of one-way analysis of variance (one-way ANOVA) and Multivariate analysis of variance (MANOVA) to determine statistically significant differences between the means of different groups (e.g., geographical regions, species, catch trends) of transboundary species and their contribution to catch and fishing revenue^68,69^. We used the standard *R* functions for the ANOVA and MANOVA after testing for assumptions. In cases where the ANOVA assumptions were not clearly met, we ran the non-parametric version Kruskal–Wallis Rank Sum Test to confirm results^70^.

### Key uncertainties

We have identified four key uncertainties in the method utilized that may affect the estimation of transboundary species. Firstly, as we ran the analysis at the species level due to limited spatial-specific data on species sub populations (stocks), we are not able to identify transboundary stocks within EEZs. While we acknowledge that a species could have multiple stocks within an EEZ, many fisheries within a country or EEZ are managed at the species instead of stock level. Also, recent research suggests strong connectivity between stocks, even when separated by thousands of kilometers^18^ providing additional ecological ground for our analysis^20,21^. However, it is important to acknowledge that there is considerable uncertainty associated in determining levels of connectivity across time and space for marine populations at different life stages, from larvae^22^ to adults^23^. This is of special concern, but-not-limited-to^23^, highly migratory species whose ranges span multiple jurisdictions and the high seas, such as tunas^71^, billfishes^72^ and sharks^73^ challenging management decisions based on meta-populations^71^. Here, we limited the definition of “transboundary” to include species spanning only adjacent countries (e.g., the USA and Canada), excluding countries that were separated by another nation (e.g., Canada and Mexico) and/or the high seas (e.g., Canada and Spain). Consequently, our results likely provide a conservative estimate of transboundary species, as we did not cover all marine taxa in the world^38^ and only analyzed species present in all four data sources. Nevertheless, our results are representative of a substantial proportion of the world’s marine catches and revenue from economically important marine fisheries. Secondly, the predicted species’ distribution is affected by the uncertainties of the environmental data and models used for such predictions. Structural differences within Earth System Models result in variations of oceanic conditions, which undoubtedly affect the ENM-Nereus. We averaged results from the three models in an effort to capture the structural variation across models. Natural climate variability is a major driver of marine species’ distributions, potentially removing a species from an EEZ for long periods (e.g., Anchovies and Sardines “regimes” in the Eastern Pacific are strongly influenced by water temperature decal oscillations^74^). Thus, a species’ distribution can potentially be reduced or shifted in such a way that it only covers one EEZ until oceanic conditions change again and the associated species’ distribution expands. To account for such climate variability, we derived the ENM-Nereus results as an average of oceanic conditions between 1970 and 2000. This is not an issue for the SDM-SAU as it does not directly require environmental variables^50^. Thirdly, we assumed that, if the *Sea Around Us* reconstructed data recorded a species as caught in any given grid cell, then the species was present within that grid cell. While catch data are not exempt of uncertainty, in most cases, differences between the *Sea Around Us* and the FAO self-reported data are smaller towards the end of the time series^52^. Thus, we limited the catch data reference period in our analyses to between 2005 and 2014, the last ten years of data available. Likewise, the spatial allocation of the catch is subjected to imprecision, mainly produced by differences in the spatial scale of the original data and the method employed by the *Sea Around Us*. Finally, this study’s results only considered species for which all datasets agreed on presence and had and *Area Index* of at least 25% (e.g., the species shared distribution was at least 25% in each EEZ). Therefore, again, our approach presents a relatively conservative estimate of the number of transboundary species in the world. Using a smaller *Area Index* will result in more transboundary species (Supplementary Fig. S2).

## Supplementary information

Supplementary Information.

## Data Availability

The *Sea Around Us* datasets used are publicly available at https://www.seaaroundus.org/, the occurrence dataset is all publicly available (see Methods), the ENM-Nereus data can be accessed upon request to G.R. The processed results and code are available at https://github.com/jepa/FishForVisa.

## References

[1] Hutchinson GE (1957). Concluding remarks. Cold Spring Harbor Symp. Quant. Biol..

[2] Nelson JS, Grande TC, Wilson MVH (2016). Fishes of the World.

[3] Song AM, Scholtens J, Stephen J, Bavinck M, Chuenpagdee R (2017). Transboundary research in fisheries. Mar. Policy.

[4] Fredston-Hermann A, Gaines SD, Halpern BS (2018). Biogeographic constraints to marine conservation in a changing climate. Ann. N. Y. Acad. Sci..

[5] Østhagen A (2020). Maritime boundary disputes: what are they and why do they matter?. Mar. Policy.

[6] United Nations. United Nations Convention on the Law of the Sea (UNCLOS)—Part V. (1986).

[7] Munro, G., Van Houtte, A. & Willmann, R. *The** Conservation and Management of Shared Fish Stocks: Legal and Economic Aspects*. FAO Fisheries Technical Paper No. 456. Food and Agriculture Organization of the United Nations, Rome (2004).

[8] Miller, K. & Munro, G. *Cooperation and Conflicts in the Management of Transboundary Fishery Resources*. (Proceeding of the Second World Conference of the Second World Congress of the American; European Associations of Environmental; Resource Economics, 2002).

[9] Englander G (2019). Property rights and the protection of global marine resources. Nature Sustainability.

[10] Spijkers J, Boonstra WJ (2017). Environmental change and social conflict: the northeast Atlantic mackerel dispute. Reg. Environ. Change.

[11] Pinsky ML (2018). Preparing ocean governance for species on the move. Science.

[12] Miller KA, Munro GR, Sumaila UR, Cheung WWL (2013). Governing marine fisheries in a changing climate: a game-theoretic perspective. Can J Agric Econ.

[13] United Nations. *Agreement for the Implementation of the Provisions of the United Nations Convention on the Law of the Sea of 10 December 1982 Relating to the Conservation and Management of Straddling Fish Stocks and Highly Migratory Fish Stocks*. (1995).

[14] Caddy J, Hancock DA (1997). Establishing a consultative mechanism or arrangement for managing shared stocks within the jurisdiction of contiguous states. Taking stock Defining and Managing Shared Resources.

[15] Teh LSL, Sumaila UR (2015). Trends in global shared fisheries. Mar. Ecol. Prog. Ser..

[16] Diario Oficial de la Federación (DOF). Carta Nacional Pesquera. Poder Ejecutivo—Secreataría de Agricultura, Ganadería, Desarrollo Rural, Pesca (SAGARPA). *Diario Oficial de la Federación DOF,* 1–268 (2018).

[17] MAP. Dictamen de Extracción No Perjudicial (DENP) de la población de "tiburón martillo" *Sphyrna zygaena*. Oficio N. 1038–2017-PRODUCE/DGPCHDI (Tra. N. 18254–2017). *Ministerio del Ambiente, Viceministerio de Desarrollo Estratégico de los Recursos Naturales, Peru* (2017).

[18] Ramesh N, Rising JA, Oremus KL (2019). The small world of global marine fisheries: The cross-boundary consequences of larval dispersal. Science.

[19] Levin N, Beger M, Maina J, McClanahan T, Kark S (2018). Evaluating the potential for transboundary management of marine biodiversity in the Western Indian Ocean. Australas. J. Environ.Manag..

[20] Popova E (2019). Ecological connectivity between the areas beyond national jurisdiction and coastal waters: safeguarding interests of coastal communities in developing countries. Mar. Policy.

[21] Dunn DC (2019). The importance of migratory connectivity for global ocean policy. Proc. R. Soc. B: Biol. Sci..

[22] Kaplan DM (2016). Uncertainty in empirical estimates of marine larval connectivity. ICES J. Mar. Sci..

[23] Archambault B (2016). Adult-mediated connectivity affects inferences on population dynamics and stock assessment of nursery-dependent fish populations. Global Environ. Change.

[24] Cashion T (2018). Establishing company level fishing revenue and profit losses from fisheries: A bottom-up approach. Journals Plos.Org.

[25] FAO. *The State of World Fisheries and Aquaculture: Meeting the Sustainable Development Goals*. 1–227 (2018).

[26] UNDP. *Chile and Peru sign Landmark Agreement to Sustain world’s Largest Single Species Fishery* (2016).

[27] NOAA FIsheries. *Bilateral Agreement Between the United States and Russia* (2019).

[28] Kleisner, K. & Pauly, D. Stock-Status Plots of Fisheries for Regional Seas. in *The State of Biodiversity and Fisheries in Regional Seas* (eds. Christensen, V., Lai, S., Palomares, M. L. D., Zeller, D. & Pauly, D.) 37–40 (The Fisheries Center, University of British Columbia; Fisheries Centre Research Reports, 2011).

[29] Jensen F, Frost H, Thogersen T, Andersen P, Andersen JL (2015). Game theory and fish wars: the case of the Northeast Atlantic mackerel fishery. Fisheries.

[30] Munro GR (2015). The management of shared fishery resources under extended jurisdiction. Mar. Resour. Econ..

[31] Eide A, Heen K, Armstrong C, Flaaten O, Vasiliev A (2013). Challenges and successes in the management of a shared fish stock—the case of the Russian-Norwegian barents sea cod fishery. Acta Borealia.

[32] Sumaila, U. R., Ninnes, C. & Oelofsen, B. Management of Shared Hake Stocks in the Benguela Marine Ecosystem. In *Papers presented at the norway-fao expert consultation on the management of shared fish stocks,* 143–159 (2003).

[33] Clark, C. W. Restricted Access to Common-Property Fishery Resources: A Game-Theoretic Analysis. In *Dynamic optimization and mathematical economics*, 117–132 (Springer, Boston, MA, 1980).

[34] Spijkers J (2019). Global patterns of fisheries conflict: forty years of data. Global Environ. Change.

[35] Oremus KL (2020). Governance challenges for tropical nations losing fish species due to climate change. Nat. Sustain..

[36] Palacios-Abrantes, J., Rashid Sumaila, U. & Cheung, W. W. L. Challenges to transboundary fisheries management in North America under climate change. *Ecol. Soc. *(in press).

[37] Sumaila, U. R., Palacios-Abrantes, J. & Cheung, W. W. L. Climate change, shifting threat points and the management of transboundary fish stocks. *Ecol. Soc. *(in press). 10.1111/gcb.16058 PMC9302671 35040239

[38] Reygondeau, G. Current and future biogeography of marine exploited groups under climate change. In *Predicting Future Oceans Sustainability of Ocean and Human Systems Amidst Global Environmental Change* (eds. Cheung, W. W. L., Ota, Y. & Cisneros-Montemayor, A. M.) 87–99 (2019).

[39] Schill SR (2015). No reef is an island: integrating coral reef connectivity data into the design of regional-scale marine protected area networks. PLoS ONE.

[40] Perez AU, Schmitter-Soto JJ, Adams AJ, Heyman WD (2019). Connectivity mediated by seasonal bonefish (Albula vulpes) migration between the Caribbean Sea and a tropical estuary of Belize and Mexico. Environ. Biol. Fishes.

[41] Cisneros-Montemayor AM, Pauly D, Weatherdon LV, Ota Y (2016). A Global estimate of seafood consumption by coastal indigenous peoples. PLoS ONE.

[42] Hanich Q (2018). Small-scale fisheries under climate change in the Pacific Islands region. Mar. Policy.

[43] Cabral RB, Geronimo RC (2018). How important are coral reefs to food security in the Philippines? Diving deeper than national aggregates and averages. Mar. Policy.

[44] Zeller D (2016). Still catching attention: Sea Around Us reconstructed global catch data, their spatial expression and public accessibility. Mar. Policy.

[45] Thuiller W, Lafourcade B, Engler R, Araújo MB (2009). BIOMOD—a platform for ensemble forecasting of species distributions. Ecography.

[46] Phillips SJ, Anderson RP, Schapire RE (2006). Maximum entropy modeling of species geographic distributions. Ecol. Model..

[47] Beaugrand G, Lenoir S, Ibanez F, Manté C (2011). A new model to assess the probability of occurrence of a species, based on presence-only data. Mar. Ecol. Prog. Ser..

[48] Asch RG, Cheung WWL, Reygondeau G (2018). Future marine ecosystem drivers, biodiversity, and fisheries maximum catch potential in Pacific Island countries and territories under climate change. Mar. Policy.

[49] Close, C. *et al.* Distribution ranges of commercial fishes and invertebrates. In *Fisheries Centre Research Reports. Fishes in Databases and Ecosystems* (eds. Palomares, M. D., Stergiou, K. I. & Pauly, D.) 27–37 (2006).

[50] Pauly D, Zeller D (2016). Global Atlas of Marine Fisheries.

[51] Kaschner, K. *et al.* AquaMaps: Predicted range maps for aquatic species www.aquamaps.org (2016).

[52] Pauly, D. & Zeller, D. Catch reconstructions reveal that global marine fisheries catches are higher than reported and declining. *Nat. Commun. 7:10244,*1–9 (2019). 10.1038/ncomms10244 PMC4735634 26784963

[53] Pebesma, E. *et al.* Package sf; Simple Features for R. **R (>= 3.3.0)**, (2018).

[54] Tai TC, Cashion T, Lam VWY, Sumaila UR (2017). Ex-vessel fish price database: disaggregating prices for low-priced species from reduction fisheries. Front. Mar. Sci..

[55] Sumaila, U. R., Teh, L., Zeller, D. & Pauly, D. The global ex-vessel fish price database. In Catch Reconstructions: Concepts, Methods and Data Sources (eds. Pauly D., & Zeller, S.) www.searoundus.org (2015).

[56] Grainger, R. J. R. & Garcia, S. M. *Chronicles of Marine Fishery Landings (1950 1994) Trend Analysis and Fisheries Potential* (1996).

[57] Pauly D, Hilborn R, Branch TA (2013). Fisheries: does catch reflect abundance?. Nature.

[58] Branch TA (2008). Not all fisheries will be collapsed in 2048. Mar. Policy.

[59] Dowle, M. *et al.* Package data.table; Extension of ‘data.frame‘. **R (>= 3.1.0)**, MPL–2.0 | file LICENSE (2019).

[60] Firke, S., Haid, C., Knight, R. & Denney, B. Package janitor; Simple tools for examining and cleaning dirty data. **R (>= 3.1.2)**, (2018).

[61] Ram, K., Wickham, H., Richards, C. & Baggett, A. Package wesanderson; A Wes Anderson Palette Generator. **R (>= 3.0)**, MIT + file LICENSE (2018).

[62] Boettiger, C., Chamberlain, S., Lang, D. T. & Wainwright, P. Package rfishbase; R Interface to ’FishBase’. **R (>= 3.0)**, (2019).

[63] Bengtsson, H., Jacobson, A. & Riedy, J. Package R.matlab: Read and Write MAT Files and Call MATLAB from Within R. **R ( 2.14.0)**, LGPL–2.1 | LGPL–3 (2018).

[64] Pebesma, E. *et al.* Package sp; Classes and methods for Spatial Data. **R ( 3.0.0)**, GPL–2 | GPL–3 (2019).

[65] Wickham, H. Package tidyverse; Easily Install and Load the ’Tidyverse’. **R (3.5.0)**, MIT + file LICENSE (2017).

[66] De Queiroz, G. *et al.* Package tidytext; Text Mining using ’dplyr’, ’ggplot2’, and Other Tidy Tools. **R ( 2.10)**, MIT (2019).

[67] Zeileis, A., Grothendieck, G., Ryan, J. A., Ulrich, J. M. & Andrews, F. Package zoo; S3 Infrastructure for Regular and Irregular Time Series (Z’s Ordered Observations). **R (>= 3.1.0)**, GPL–2 | GPL–3 (2019).

[68] Chambers JM, Freeny AE, Heiberger RM, Chambers JM, Hastie TJ (1992). Analysis of Variance; Designed Experiments. Statistical models in s.

[69] Krzanowski WJ (1990). Principles of Multivariate Analysis.

[70] Hollander M, Wolfe DA (2013). Nonparametric Statistical Methods.

[71] Moore BR (2020). Defining the stock structures of key commercial tunas in the Pacific Ocean I: current knowledge and main uncertainties. Global Environ. Change.

[72] Sepulveda CA, Wang M, Aalbers SA, Alvarado-Bremer JR (2019). Insights into the horizontal movements, migration patterns, and stock affiliation of California swordfish. Fish. Oceanogr..

[73] Vandeperre F (2014). Movements of Blue Sharks (*Prionace glauca*) across Their Life History. PLoS ONE.

[74] Chavez FP, Ryan J, Lluch-Cota SE, Niquen CM (2003). From anchovies to sardines and back: multidecadal change in the Pacific Ocean. Science.

