# Supplementary material for: The transboundary nature of the world’s exploited marine species
Source: Sci Rep. 2020 Oct 21;10:17668. doi: 10.1038/s41598-020-74644-2 (PMC7578035; doi:10.1038/s41598-020-74644-2)
Supplement: Supplementary file 1 — Supplementary Information. [file 41598_2020_74644_MOESM1_ESM.pdf]

Supplementary information for The transboundary nature of the world's exploited marine species

Authors: Juliano Palacios-Abrantes, Gabriel Reygondeau, Colette C.C. Wabnitz and William W.L. Cheung

Supplementary Figures

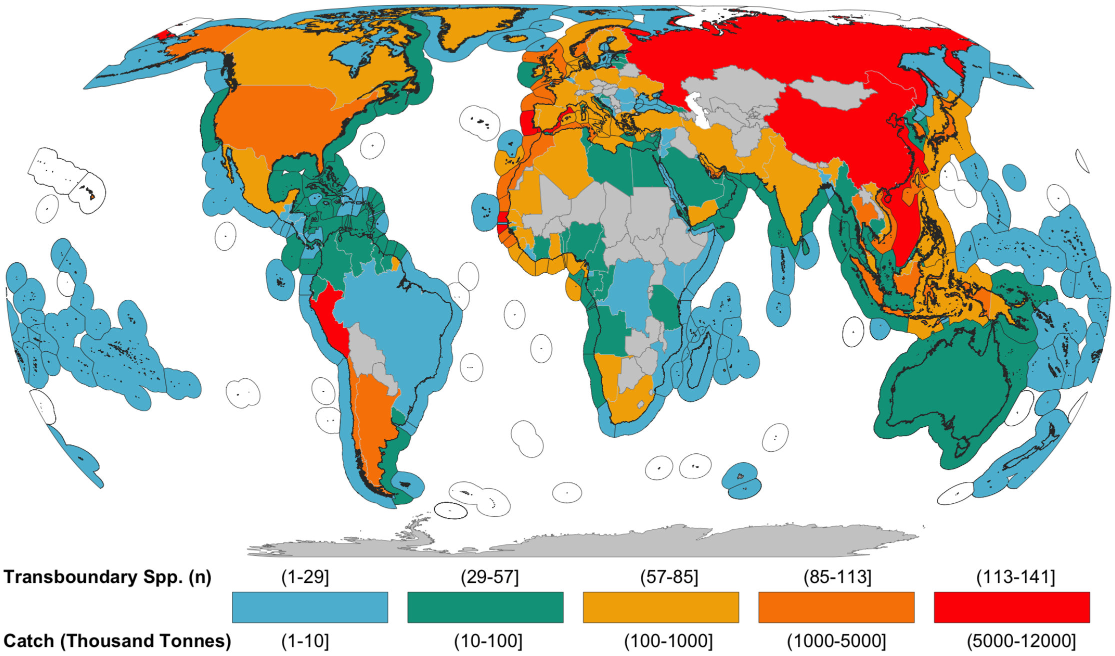

Figure S 1: Number of transboundary species per EEZ and their contributions to countries' EEZ catch. The number of species is displayed in the EEZ while catch (in thousand tonnes) is displayed in the land polygon. Figure created using R version 3.5.2. Land and EEZ shapefiles from Natural Earth (www.naturalearthdata.com) and Sea Around Us (www.seaaroundus.org), respectively.

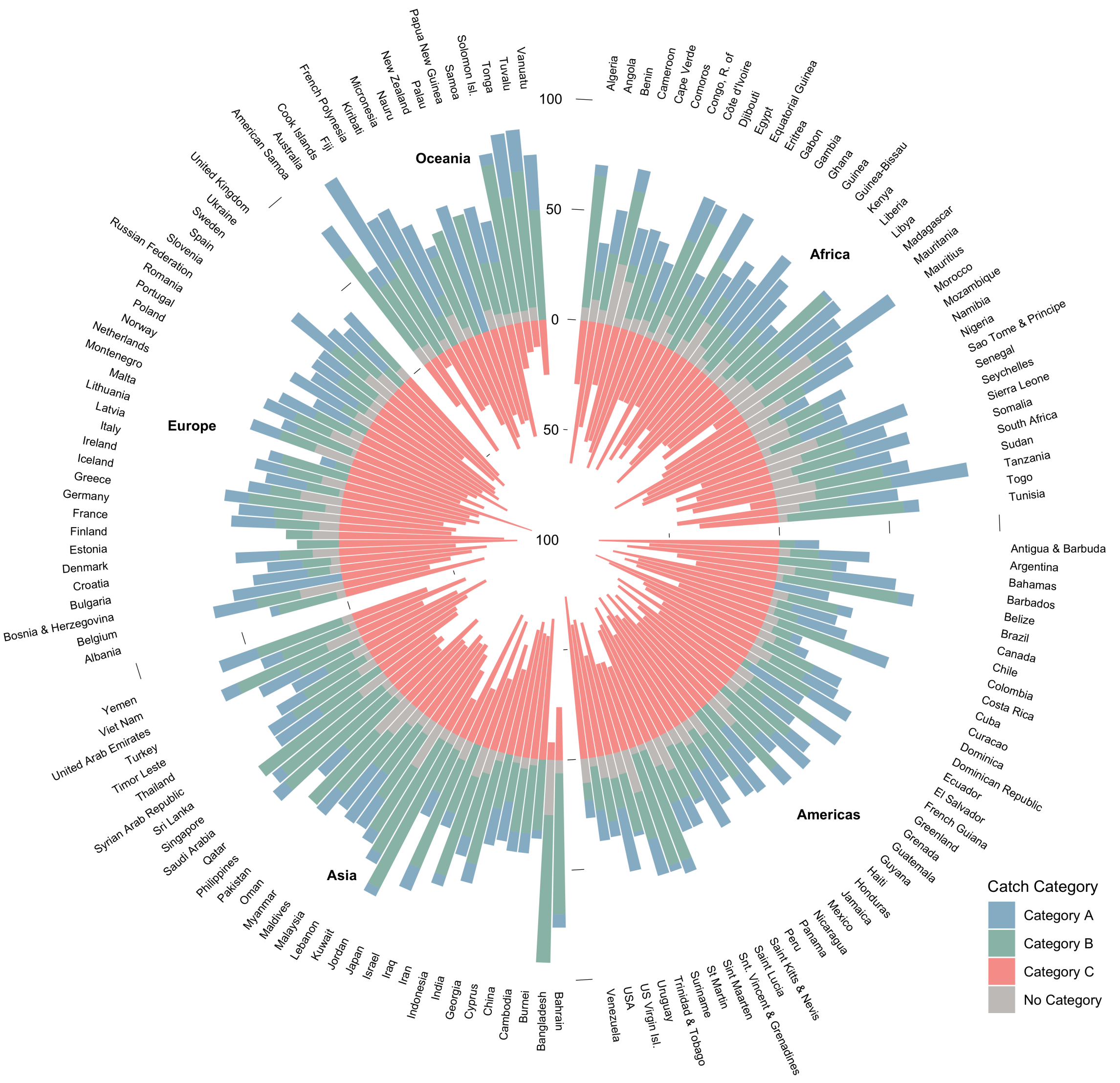

Figure S 2: Percentage of transboundary species by catch trend and EEZ. Category A, Increasing; Category B, Constant; Category C, Decreasing. "No Category" reflects species with less than 10 years of catch data and/ or less than 5 consecutive years of catch data. Figure created using R version 3.5.2.

The number of grid cells in which a species is present (*Area Index*) is expected to directly influence whether or not a species is considered transboundary. Therefore, we tested the sensitivity of our results to the *Area Index* by estimating the number of transboundary species along a gradient of values (Supplementary Fig. S3). As expected, using a more relaxed value (e.g. the species is present within 10% or more of the EEZ) will result in a greater number of transboundary species (median of 40 EEZs shared per species), while a more restrictive value (e.g., 50%) results in a median estimate of 4. The current analysis utilized a 25% *Area Index* threshold (Supplementary Fig. S3 - darker histogram).

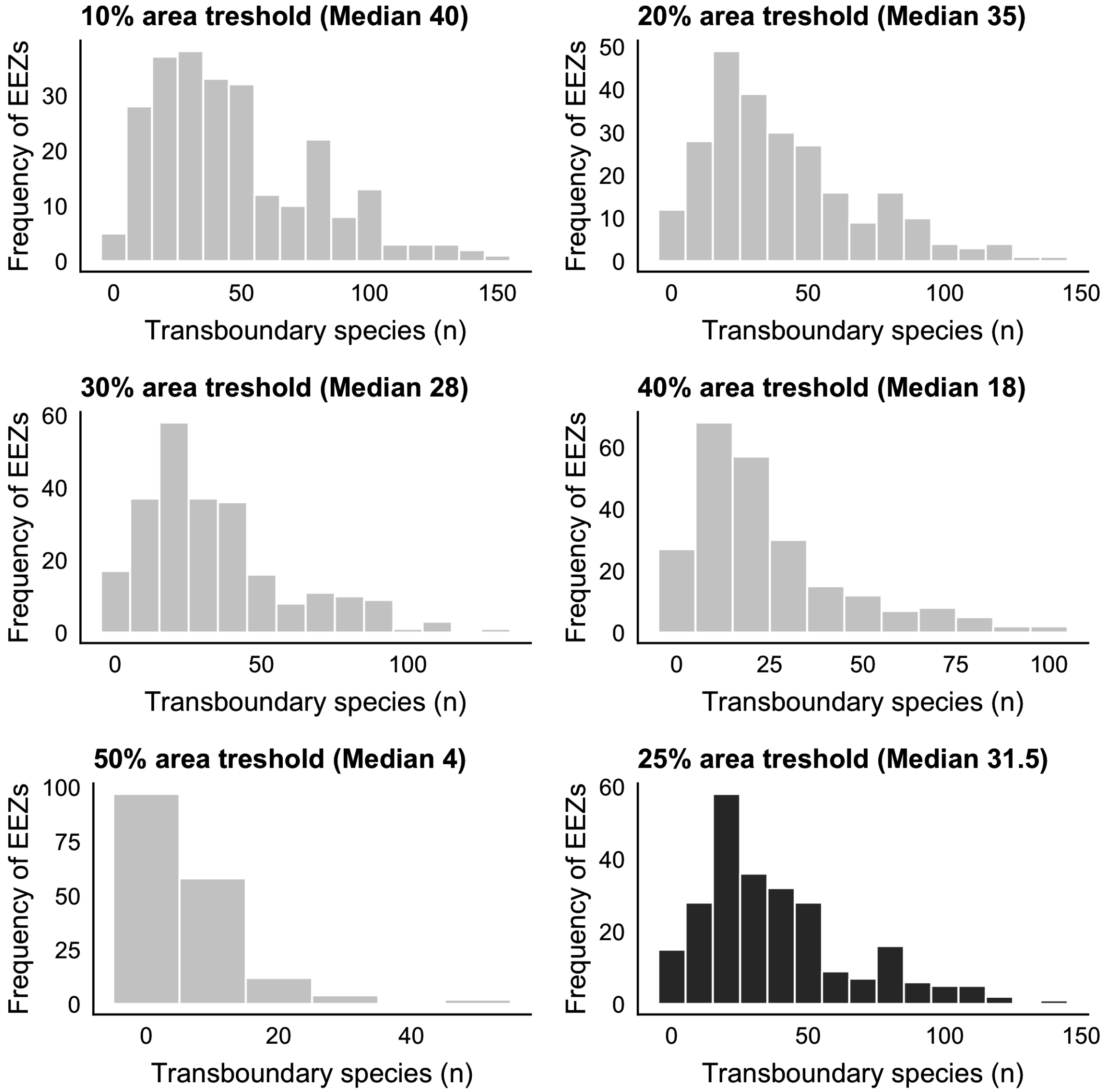

Figure S 3: Histogram of number of transboundary species using different Area Index threshold values. The darker histogram represents the threshold (25%) used in the present analysis. Figure created using R version 3.5.2.

Supplementary Tables

Table S 1: Size of all the EEZs in each sub region determined by the United Nations

UN sub region	Area of EEZs (Km^2)
Northern Africa	1423774
Western Asia	1744784
Western Europe	2018481
Southern Europe	2484760
Southern Asia	4206953
Northern Europe	6401144
Eastern Asia	7329899
Melanesia	7525195
Eastern Europe	7777970
Micronesia	8313054
Polynesia	9577373
South-eastern Asia	11098246
Australia and New Zealand	12282183
Sub-Saharan Africa	12349817
Latin America and the Caribbean	20723420
Northern America	22081778

Table S 2: Average ± (standard deviation) number of shared and non-shared transboundary species per EEZ for each catch trend category

Category	Mean shared	Mean not-shared
Category A	8.19 ± (7.94)	3.95 ± (3.77)
Category B	14.16 ± (13.61)	6.86 ± (7.76)
Category C	25.12 ± (20.4)	12.45 ± (14.61)

Translated Abstacts

Español

Es frecuente que los límites de las herramientas de manejo ambiental y la distribución de especies no coincidan, como sucede en el caso de especies marinas que cruzan múltiples Zonas Económicas Exclusivas (ZEE). Este tipo de movimiento representa un desafío para el manejo pesquero, ya que las políticas públicas tienden a enfocarse en el nivel nacional; sin embargo, es necesario establecer colaboraciones internacionales para maximizar los beneficios ecológicos, sociales y económicos a largo plazo obtenidos con la captura de especies marinas compartidas. En este artículo se combinó la distribución de especies con la delimitación espacial de las ZEE para identificar el número de especies marinas explotadas comercialmente que son compartidas por naciones vecinas (especies transfronterizas). Los resultados revelaron que el 67% de las especies analizadas pertenecen a esta categoría (n = 633). Entre 2005 y 2014, las pesquerías que tenían como objetivo estas especies y que operaban dentro de las ZEE mundiales capturaron un promedio de 48 millones de toneladas por año, lo que equivale a un promedio de 77 mil millones de dólares en ingresos anuales por pesca. Durante este tiempo, más del 90% de las capturas y beneficios económicos de algunos países fue resultado de unos pocos recursos compartidos. Así mismo, se demuestra que las capturas de especies transfronterizas están disminuyendo a un ritmo mayor que las no transfronterizas. Este estudio tiene implicaciones directas para el manejo pesquero de dichas especies, destacando la necesidad de fortalecer una cooperación internacional eficiente y equitativa.

Português

É frequente que os limites dos instrumentos de gestão de recursos naturais e a distribuição das espécies não coincidam como acontece no caso das espécies marinhas que cruzam múltiplas Zonas Econômicas Exclusivas (ZEE). Este tipo de movimento representa um desafio para a gestão pesqueira, já que as políticas públicas tendem a ser focadas ao nível nacional; portanto, colaborações internacionais precisam ser estabelecidas para maximizar os benefícios ecológicos, sociais e econômicos de longo prazo alcançados com a captura de espécies marinhas compartilhadas. Neste artigo, a distribuição das espécies foi combinada com a delimitação espacial de ZEEs para identificar o número de espécies marinhas exploradas comercialmente que são compartilhadas por nações vizinhas (espécies transfronteiriças). Os resultados revelaram que 67% das espécies analisadas pertencem a essa categoria (n = 633). Entre 2005 e 2014, as pescarias que operavam nas ZEEs globais que visam essas espécies capturaram uma média de 48 milhões de toneladas por ano, o que equivale a uma média de $ 77 bilhões em receitas anuais de pesca. Durante este tempo, mais de 90% das capturas e benefícios econômicos de alguns países foi resultado de alguns recursos compartilhados. Da mesma forma, se mostra que as capturas de essas espécies estão diminuindo a um ritmo maior do que as das não transfronteiriças. Este estudo tem implicações diretas para a gestão da pesca de espécies referidas, destacando a necessidade de fortalecer uma cooperação internacional eficiente e equitativa.

Français

Dans la grande majorité des cas, les limitations spatiales des zones internationales de pêche et la distribution des espèces ne s'alignent pas. C'est particulièrement le cas pour les espèces à large distribution qui occupent et migrent à travers plusieurs zones économiques exclusives (ZEE). Les mouvements de ces espèces représentent donc un défi pour la gestion durable de leurs stocks. En effet, l'évaluation des stocks est actuellement effectuée à une échelle nationale alors qu'une approche internationale est préconisée afin de maximiser les avantages écologiques, sociaux et économiques liés à ces espèces. Dans cette étude, nous avons combiné, à l'échelle mondiale, la distribution des espèces marines exploitées ainsi que la distribution des ZEE afin d'identifier le nombre d'espèces marines partagées entre pays avoisinants. Nos résultats démontrent que 67% des espèces considérées sont transfrontalières (n = 633). Entre 2005 et 2014, les pêcheries ciblant ces espèces dans les ZEE ont capturé, en moyenne, 48 millions de tonnes par an, ce qui équivaut à 77 milliards USD en revenus moyen par an. Dans certains cas, plus de 90% des captures et revenus sont attribuables à un nombre restreint

d'espèces partagées. Notre analyse suggère par ailleurs que les captures d'espèces transfrontalières marquent un déclin plus marqué que celles des autres espèces . Dans un contexte de gestion écosystémique des pêches, les résultats de notre étude soulignent l'importance d'une coopération internationale efficace et équitable afin d'atteindre une pêcherie globale durable
